# Maternal prenatal mental health and substance Use: a review of prevalence, mechanisms, outcomes, and interventions

**DOI:** 10.3389/fgwh.2026.1744391

**Published:** 2026-06-17

**Authors:** Pamela Schuetze

**Affiliations:** Department of Psychology, Buffalo State University, Buffalo, NY, United States

**Keywords:** developmental outcomes, maternal mental health, perinatal mood and anxiety disorders, prenatal substance use, screening and treatment

## Abstract

Maternal mental health during pregnancy interacts with substance use, exacerbating risks for adverse developmental outcomes in offspring. This review synthesizes evidence on the prevalence and co-occurrence of prenatal mental health and substance use, mechanisms linking the two, consequences for maternal and child health, screening and treatment strategies, and system-level policy implications. Key themes include the high prevalence of perinatal mood and anxiety disorders, elevated rates of substance use among pregnant women with mental illness, synergistic effects of combined exposures on developmental outcomes, and substantial barriers to appropriate treatment. Effective practices emphasize universal screening, trauma-informed and nonpunitive approaches, and integrated behavioral health and substance use treatment in perinatal settings. Future research should identify causal pathways from prenatal risk to child outcomes using longitudinal designs with diverse samples, examine culturally responsive interventions to mitigate disparities, and should test scalable models that provide both mental health and substance use services in perinatal care.

## Introduction

Maternal mental health and substance use during pregnancy are critical public health concerns with significant implications for both the mother and the child. Pregnancy is a time during which women are particularly vulnerable to mood and anxiety disorders. Epidemiological research indicates that pregnancy mental health concerns and substance use frequently co-occur, exacerbating risks for adverse maternal and developmental outcomes (1, 2). In addition to their negative impact on maternal well-being (3), as highlighted by the developmental origins of health and disease hypothesis, these comorbidities may alter fetal neurodevelopment through biological pathways, potentially leading to long-term developmental outcomes (4). Furthermore, mental health issues and substance use frequently continue into the postnatal period and have the potential to adversely impact child development through environmental pathways. Despite growing recognition of the interplay between maternal psychopathology and substance use, research on identifying shared mechanisms and protective factors that may mitigate these risks remains sparse.

Mental health disorders are among the most common complications during pregnancy and childbirth and are associated with negative outcomes for children beginning at birth (5–7). Research consistently indicates that maternal prenatal and postnatal mental health impact long-term developmental outcomes, including heightened vulnerability to adverse neurodevelopmental and behavioral outcomes from infancy through adolescence (7). For instance, maternal anxiety experienced before or shortly after birth has been associated with greater infant negative affect and internalizing problems in childhood (8, 9). The impact of maternal mental health on children likely depends on their nature, timing, and intensity of the psychological symptoms, and may be amplified by additional stressors such as substance use or low socioeconomic status (10).

## Prevalence

Estimates of the prevalence of maternal psychopathology vary by setting, measurement method, and the specific disorder examined, but pooled estimates typically indicate that roughly 10%–25% of pregnant women experience clinically significant depressive or anxiety symptoms, with higher rates reported in some large urban or high-risk samples and during population stressors such as the COVID-19 pandemic (11). Depression affects approximately 10%–20% of pregnant women and is associated with adverse perinatal outcomes such as preterm birth, low birth weight and socioemotional development during infancy (12). In addition, approximately 1 in 5 pregnant women experiences clinically significant levels of anxiety (13). Recent large-scale analyses continue to underscore that perinatal mental disorders are common and frequently underdiagnosed (11).

Substance use in pregnancy remains a significant public health concern. Recent epidemiological research suggests that use of substances during pregnancy is not rare. In fact, population estimates suggest that in recent years roughly 10%–20% of pregnant people report some substance use, with variation by substance type and by sociodemographic group. Research has identified tobacco, alcohol and cannabis as the most commonly used substances in pregnancy, with cocaine and opioids used less frequently but associated with significant clinical risks for the exposed offspring. For example, data from the National Survey on Drug Use and Health (NSDUH) show that approximately 8.4% of pregnant women (ages: 15–44) reported alcohol use in the past month (14) whereas approximately 0.9% of pregnant women report using opioids within the past month (15) and about 5.1% of pregnant women report the use of multiple substances during pregnancy (16).

Importantly, psychological disorders and substance use often co-occur. Although estimates of comorbid substance use and mental health issues among pregnant women depend on how substance use and mental health are defined, it is estimated that approximately 1.8%–5.3% of pregnant women have a psychiatric and substance use comorbidity (17) although these estimates likely vary substantially by trimester of assessment, specific substances assessed, geographic area, method of assessing substance use and sociodemographic factors.

## Mechanisms linking prenatal mental health and substance use

Several pathways explain the frequent comorbidity of prenatal mental health problems and substance use. First, self-medication and negative reinforcement models propose that individuals may use substances to attenuate distressing mood or anxiety symptoms (18). Second, shared vulnerability factors (genetic predisposition, early adversity, sociodemographic risk, intimate partner violence, and chronic stress) increase risk for both psychological disorders and substance use. Third, neurobiological changes in stress-response systems (e.g., hypothalamic–pituitary–adrenal axis dysregulation) and reward circuitry can increase both mood dysregulation and susceptibility to substance reinforcement (19). Finally, service-level and structural barriers, including stigma, fear of punitive consequences, lack of integrated services, and inadequate insurance coverage, reduce help-seeking and treatment engagement, allowing co-morbidity to persist during pregnancy (20).

## Maternal and neonatal outcomes

Maternal mental disorders and substance use in pregnancy are each independently associated with adverse perinatal outcomes (e.g., preterm birth, low birth weight, hypertensive disorders), but the combination often confers synergistic risk (21). Beyond obstetric complications, pregnant women with co-occurring mental illness and substance use face higher rates of postpartum psychiatric morbidity, especially postpartum depression and anxiety, elevated parenting stress, and increased likelihood of socioeconomic instability that can complicate caregiving after delivery.

Prenatal exposure to substances is associated with a range of adverse neonatal outcomes that vary by substance, dose and timing. These outcomes include low birth weight, small-for-gestational-age status, preterm delivery, and for opioid-exposed infants, neonatal opioid withdrawal [neonatal abstinence syndrome; (21)]. Longer-term developmental outcomes documented in reviews include increased risk for attentional and executive function deficits, externalizing behavior problems, and heightened need for pediatric and special services; however, disentangling the direct teratologic effects of substances from postnatal environmental confounds (e.g., caregiving stress, socioeconomic disadvantage) remains a central methodological challenge (21).

## Screening

Because of the comorbidity of mental health problems and substance use during pregnancy, universal screening for both prenatal mental health concerns and substance use during prenatal care is recommended by many professional organizations [e.g., (22)]. Prenatal care visits are an accessible point of contact for identification and early intervention and evidence supports the feasibility and effectiveness of routine, universal screening, when implemented with validated tools, clear referral pathways, staff training, and safeguards against punitive consequences (23). Screening should be brief, standardized, culturally sensitive, and integrated into routine obstetric workflows. Examples of validated screening tools include the Edinburgh Postnatal Depression Scale (EPDS) for mood symptoms and 4Ps, TWEAK, or single-item screening with follow-up for substance use. Importantly, screening programs should incorporate nonjudgmental communication, confidentiality protections, and linkage to treatment. Unfortunately, barriers such as fear of stigma, lack of referral systems and punitive reporting laws stand in the way of pregnant women seeking and receiving necessary services (20).

## Treatment and models of care

Treatment models that simultaneously address mental health and substance use services within prenatal care show promise for impacted mothers. These models range from embedding behavioral health clinicians embedded within obstetrics clinics to multidisciplinary programs offering specialized perinatal substance use treatment, medication-assisted treatment (for opioid use disorder), psychotherapy (e.g., cognitive-behavioral therapy), and psychosocial supports (housing, childcare, legal aid). Systematic reviews of such treatment programs document increased engagement and retention in treatment, reductions in substance use, and improved maternal mental health outcomes, although randomized trials with long-term child outcomes remain limited. It is important to note that the efficacy of treatment programs for pregnant women is dependent on the inclusion of trauma-informed care principles given the high prevalence of past trauma in populations with substance use disorders (24).

Pharmacotherapy interventions for psychological or substance use disorders during pregnancy are available but require careful risk–benefit analyses (25). For many pregnant women with moderate to severe depression or anxiety, selective serotonin reuptake inhibitors (SSRIs) or other psychotropic medications may be clinically indicated given that untreated severe mental illness also carries risks to both mother and fetus. For opioid use disorder, medication-assisted treatment with methadone or buprenorphine during pregnancy is the evidence-based standard of care and reduces overdose risk and improves prenatal care engagement. For other substance use disorders (e.g., alcohol use disorder), pharmacologic options are more limited during pregnancy and emphasize psychosocial interventions and harm reduction where complete cessation is not immediately achievable. In all cases, collaborative decision-making with obstetric, psychiatric, and addiction specialists is recommended.

Other treatment approaches, including brief motivational interventions, cognitive-behavioral strategies, peer support, and home-visiting programs, have demonstrated variable but promising effects in reducing substance use and improving maternal mental health in perinatal populations (25). However, for any treatment to be effective, social risk factors including housing insecurity, food insecurity, and intimate partner violence must be addressed alongside clinical interventions. Programs that combine behavioral treatment with social support services tend to achieve better engagement and sustained outcomes (14).

Despite their importance, screening and intervention for substance use and mental disorders in pregnancy occur within a fraught ethical and legal context. In some jurisdictions, disclosure of prenatal substance use can trigger child protective services involvement or criminalization, deterring pregnant women from seeking care (26). However, research has shown that such consequences are ineffective in reducing rates of substance use during pregnancy (26). Rather, nonpunitive, voluntary, and supportive approaches that prioritize treatment access over surveillance are associated with better disclosure, engagement, and maternal and child outcomes (27). Racial and socioeconomic disparities for psychological and substance use disorders are also pervasive among pregnant women. Marginalized groups often face higher rates of severe perinatal mental illness, greater barriers to care, and more punitive legal responses (28). Thus, strategies that focus on equity and cultural competence, and policy reforms that remove criminalizing penalties for prenatal substance use are critical to ensure access to treatment and to reduce harm.

## Discussion

Current research addressing the co-morbidity of maternal substance use during pregnancy and maternal mental health disorders is concentrated in several key areas, including the identification of co-occurring patterns and risk profiles, investigation of shared underlying mechanisms (e.g., trauma exposure, adverse childhood experiences, and stress dysregulation), and the development of integrated, interdisciplinary treatment models. Epidemiological studies increasingly use person-centered approaches to characterize subgroups at heightened risk and to examine how psychiatric comorbidities predict continued substance use across the perinatal period (29, 30). Concurrently, mechanistic research highlights the central role of trauma and social determinants in shaping both substance use and mental health trajectories (31). Intervention-focused studies emphasize integrated care models that combine obstetric, behavioral health, and social services, as well as early screening and prevention strategies delivered during pregnancy as a critical window for engagement (32). Additionally, there is growing attention to health disparities and structural barriers, with researchers advocating for culturally responsive, equity-oriented approaches to improve access and outcomes for vulnerable populations (33).

Despite advances in understanding the negative impact of maternal mental health problems and substance use during pregnancy, important gaps in knowledge remain. In particular, the causal pathways linking maternal mental health, substance use, and child outcomes are not yet well understood, highlighting the need for prospective, longitudinal studies that include diverse cohorts and rigorous measurement of prenatal substance exposure, key mediators (e.g., physiological reactivity and caregiving environment), and long-term developmental outcomes (33, 34). These gaps are especially critical given robust evidence that prenatal substance use frequently co-occurs with depression, anxiety, and posttraumatic stress disorder, with these conditions mutually reinforcing one another and contributing to adverse maternal–infant outcomes (35, 36). Accordingly, future research should prioritize interdisciplinary approaches that elucidate underlying mechanisms—such as adverse childhood experiences, stress regulation, and social determinants—that shape both substance use and mental health trajectories (37, 38), while also expanding research on trauma-informed interventions that account for cultural context and socioeconomic disparities.

At the same time, intervention research remains limited, particularly with respect to scalable, comprehensive treatment models. Future efforts should move beyond siloed approaches toward integrated, trauma-informed care frameworks that simultaneously address psychiatric symptoms, substance use, and contextual risk factors within prenatal and postpartum systems (40). Promising strategies include embedding behavioral health services within case management and prenatal care, implementing coordinated perinatal substance use disorder programs, and promoting early identification through universal screening across healthcare settings (39, 40). In addition, emphasis on culturally responsive, equity-focused interventions and sustained postpartum support will be essential to reduce relapse risk and promote long-term maternal well-being (41). Finally, incorporating implementation science and rigorous evaluation, especially through randomized and longitudinal designs, is critical to determining how these multilevel interventions can be effectively adapted, scaled, and sustained across diverse clinical and community settings (42).

In conclusion, maternal prenatal mental health and substance use are related public health challenges with important implications for both maternal and child well-being. Fortunately, these risks can be mitigated through early detection, comprehensive and trauma-informed treatment along with supportive social policies. Scaling evidence-based, culturally responsive models of perinatal care, while removing punitive barriers to disclosure, can lead to improved outcomes for families.
